# Effectiveness of Different Methods of Interdental Hygiene in Daily Practice Among Young Adults: Protocol for a Randomized, Single-Blind Controlled Trial

**DOI:** 10.2196/85154

**Published:** 2025-12-04

**Authors:** Florence Carrouel, Romain Lan, Ina Saliasi, Denis Bourgeois, Nicolas Benech, Audrey Murat-Ringot, Stéphane Viennot

**Affiliations:** 1 Health Systemic Process (P2S) UR4129, Université Claude Bernard Lyon 1, 7 Rue Guillaume Paradin, Lyon, 69008, France, +33478785745; 2 Laboratory Anthropologie bio-culturelle, droit, éthique et santé (ADES), Centre National de la Recherche Scientifique (CNRS), Etablissement français du sang (EFS), Aix Marseille University, Marseille, France; 3 Lyon GEM Microbiota Study Group, Hospices Civils of Lyon, Lyon, France; 4 Centre de Recherche en cancérologie, Claude Bernard Lyon 1 University, Lyon, France; 5 Hospices Civils of Lyon, Lyon, France

**Keywords:** prophylaxis, oral hygiene, periodontitis, gingival inflammation, microbiota, biofilm, toothbrush

## Abstract

**Background:**

Interdental spaces are particularly susceptible to biofilm accumulation and gingival inflammation, which contribute to periodontal diseases and their systemic associations. While interdental brushes (IDBs) are recognized as the most effective method of interdental cleaning, their efficacy depends on proper adaptation to the interdental space. Calibration with a colorimetric probe may enhance their effectiveness and comfort. However, evidence directly comparing calibrated and noncalibrated IDBs, especially in young adults, a key target group for preventive strategies, remains limited. The Hygiene of Interdental Junctions in Adults (HIJA) trial was designed to address this gap.

**Objective:**

This protocol aims to compare the clinical, microbiological, and acceptability outcomes associated with calibrated versus noncalibrated IDBs in young adults, focusing on interdental inflammation, periodontal indices, and microbiota composition.

**Methods:**

The HIJA trial is a monocentric, randomized, controlled, single-blind, and parallel-arm study. Overall, 50 healthy, nonsmoking adults aged 18‐30 years will be randomized (1:1) to receive either calibrated or noncalibrated IDBs. Participants will perform daily interdental cleaning in addition to conventional toothbrushing for 3 months. The primary outcome will be the reduction in interdental inflammation, expressed as the change in bleeding on probing at 3 months. Secondary outcomes will assess differences in interdental microbiota composition (16S ribosomal RNA sequencing), periodontal indices (plaque index, gingival index, probing depth, and clinical attachment loss), and user acceptability measured through the Theoretical Framework of Acceptability questionnaire at 1, 2, and 3 months.

**Results:**

The HIJA trial will generate evidence on whether calibrated IDBs provide additional benefits over noncalibrated brushes in reducing interdental inflammation and improving oral health in young adults.

**Conclusions:**

HIJA findings could contribute to the implementation of clinical guidelines and preventive strategies for interdental hygiene in daily practice.

## Introduction

Oral health is a cornerstone of general well-being and systemic health [1-3]. Among the multiple ecological niches of the oral cavity, interdental spaces represent unique sites with minimal host defense mechanisms. Their anatomical configuration predisposes them to bacterial colonization and biofilm accumulation, which in turn promotes local inflammation and increases the risk of periodontal disease [4,5]. Periodontal diseases are strongly associated with systemic conditions, including cardiovascular disease, diabetes, adverse pregnancy outcomes, and certain cancers, making early prevention a public health priority [1-3].

The interdental microbiota plays a pivotal role in this process. A balanced microbiota contributes to oral homeostasis, while dysbiosis triggers gingival inflammation and subsequent periodontal destruction. Interdental cleaning, therefore, represents a key strategy to prevent dysbiosis [6,7]. Conventional toothbrushing cannot access interdental areas, which highlights the need for complementary interdental hygiene practices [8].

Current evidence indicates that interdental brushes (IDBs) are the most effective and widely recommended method of interdental cleaning, outperforming dental floss and wooden sticks in reducing gingival inflammation and plaque accumulation. Several meta-analyses and network analyses have confirmed their superiority, while observational studies have shown their acceptability in daily use [4,9]. However, substantial heterogeneity exists among IDBs, particularly regarding size, shape, and calibration. Calibration with a colorimetric probe ensures optimal adaptation of IDB to interdental space, thereby improving mechanical efficacy and user comfort [10].

Despite these insights, there is a paucity of clinical evidence comparing calibrated versus noncalibrated IDBs in young adults, a critical population in which preventive strategies should be implemented before the onset of irreversible periodontal damage [11]. To date, few randomized controlled trials have demonstrated that daily calibrated IDB use significantly reduces interdental inflammation and dysbiosis in young adults [6,7]. No study has yet addressed whether such calibration provides a measurable advantage in real-life daily practice compared to noncalibrated IDBs.

The Hygiene of Interdental Junctions in Adults (HIJA) trial is designed to address the following research question, formulated according to the Population, Intervention, Comparison, and Outcome framework: In healthy young adults aged 18‐30 years (Population, P), does the daily use of calibrated IDBs (Intervention, I) lead to a greater reduction in interdental inflammation compared with the daily use of noncalibrated IDBs (Comparator, C), measured as bleeding on probing (BoP) at 3 months (Outcome, O)?

## Methods

### Trial Design

This clinical study named HIJA is designed as a monocentric, randomized, controlled, 2 parallel-arm, and single-blind clinical study (ratio 1:1; Figure 1). The protocol is presented in accordance with SPIRIT (Standard Protocol Items: Recommendations for Interventional Trials) guidelines [12].

**Figure 1.**
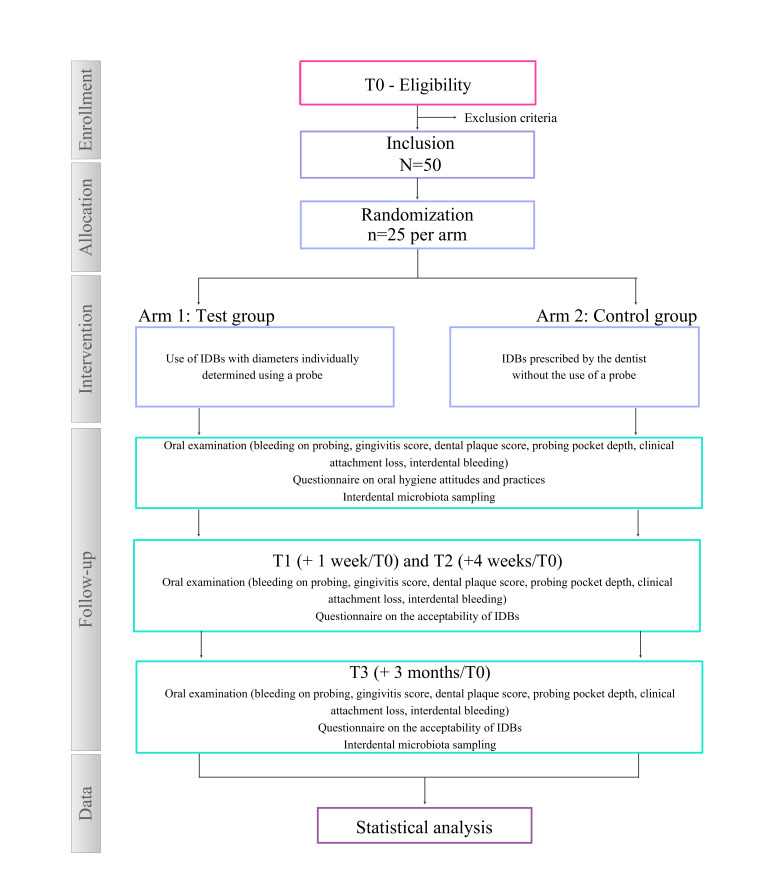
Flowchart diagram of Hygiene of Interdental Junctions in Adults study. IDB: interdental brush.

### Study Setting

The HIJA study will be conducted within the Odontology Department of the Hospices Civils of Lyon, France.

### Study Population

#### Eligibility Criteria

##### Inclusion Criteria

Eligibility criteria will be (1) participants between 18 and 30 years of age, (2) who have given their free, informed, and express written consent, (3) affiliated with a French social security scheme, and (4) living or working in Lyon or the Lyon metropolitan area.

Periodontal health will be required for inclusion, in accordance with the 2017 World Workshop classification [13], defined as the absence of gingival inflammation or active attachment loss. A reduced but stable periodontium, such as mild localized recession without probing depth ≥4 mm or generalized involvement, will be considered consistent with periodontal health and may include cases corresponding to Stage I periodontitis when such findings are localized, noninflammatory, and clinically stable.

##### Exclusion Criteria

Exclusion criteria will be (1) individuals with a tobacco addiction, (2) currently participating in another study related to oral hygiene, (3) at high risk of infective endocarditis, (4) with chronic medical conditions such as type 1 or 2 diabetes, (5) who have taken antibiotic treatment during the month preceding the start of the study, (6) pregnant or breastfeeding women, (7) deprived of their liberty by a judicial or administrative decision, (8) receiving psychiatric care, (9) admitted to a health or social care facility for purposes other than research, (10) subject to a legal protection measure (guardianship and curatorship), (11) not affiliated with a social security scheme or beneficiaries of a similar scheme, (12) with fewer than 20 natural teeth, (13) currently performing interdental hygiene and/or daily mouthwashes, (14) wearing orthodontic appliances, (15) with periodontal disease (stage II or higher according to the 2017 Chicago classification [ie, probing pocket depth, PPD ≥4 mm and/or clinical attachment loss, CAL ≥4 mm] and/or generalized, >30% of sites), active caries, or undergoing dental treatment, and (16) under judicial protection.

### Materials Description

#### Colorimetric Probe

**Figure 2.**
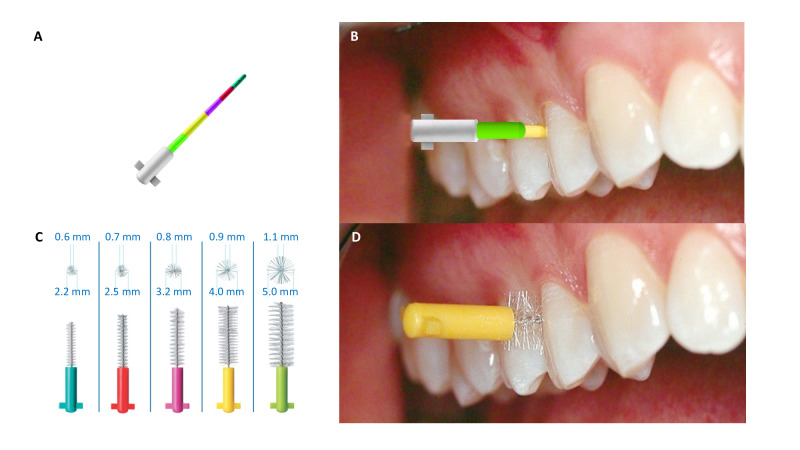
Procedure for using the colorimetric probe to determine the appropriate interdental brush size. (**A**) Colorimetric probe, (**B**) color indication (yellow) corresponding to the measured interdental space, (**C**) available interdental brushes color-coded by size, and (**D**) use of the selected interdental brush in the interdental space.

The IAP CURAPROX calibrating probe is a graduated conical instrument with a rounded tip (Figure 2A and B). The working part comprises colored bands from the point to the base corresponding to IDBs by increasing diameter. The largest section of each colored band corresponds to the cleaning efficiency diameter of the relevant brush. The nonworking part has a click-fastening joint for the attachment of a handle for easier use and access to the interproximal spaces at the back of the mouth.

Interdental diameters were coded as 1=blue, 2=red, 3=pink, 4=yellow, and 5=green. The procedure consists of introducing the IAP CURAPROX probe into the vestibular interdental space, inserting it fully, then noting the color emerging from the interdental space on the vestibular side. This corresponds to the color code of the IDB most suitable for the interdental space in question. The pressure used to place the probe tip at the base of the interdental sites was approximately 50 N/cm^2^ (0.20 gram force).

#### Interdental Brushes

The IDBs used in this study are from the CPS range of CURAPROX (Curaden; Figure 2C and 2D). These IDBs consist of cylindrical nylon fiber brushes mounted on a CURAL wire core coated with synthetic material to prevent galvanic or traumatic effects. The brushes are supplied with ergonomic plastic handles to facilitate grip and insertion in posterior sites. Each CPS pack comprises five IDBs with the following characteristics: (1) a specific color code corresponding to the brush size (Table 1); (2) an access diameter, defined by the gauge of the CURAL wire core, which determines the minimal interdental space into which the brush can be inserted; and (3) an effective cleaning diameter, defined by the length and elasticity of the synthetic bristles covering the working part of the device.

**Table 1.** Characteristics of interdental brushes used in the Hygiene of Interdental Junctions in Adults study.

Color code	Access diameter (mm)	Cleaning diameter (mm)
Blue	0.6	2.2
Red	0.7	2.5
Pink	0.8	3.2
Yellow	0.9	4
Green	1.1	5

### Interventions

In the intervention group, participants will receive IDBs with diameters individually determined by the dentist using a colorimetric probe to ensure optimal adaptation to each interdental space. In the control group, participants will receive IDBs prescribed by the dentist without calibration by a probe. In both groups, the dentist will visually assess the morphology of the interdental spaces, including the presence or partial loss of interdental papilla, to ensure that the prescribed brushes can be safely and comfortably inserted. This clinical evaluation will complement the calibration procedure in the intervention group and will guide professional judgment in the control group. All participants will be instructed to use IDBs once daily, in addition to their regular toothbrushing routine, for a duration of 3 months. They will not be allowed to use complementary techniques (dental floss, mouthwashes, etc) during the period of the study. All participants will be naive users of IDBs at baseline, as individuals already performing interdental hygiene will be excluded according to the study criteria.

Individual instructions on interdental brushing hygiene techniques will be provided by the dentist, who will first ensure that each participant is able to handle the IDBs correctly and safely, demonstrating sufficient manual dexterity and cognitive ability to perform the technique as instructed. Each participant will be provided with a logbook at the baseline visit to record daily IDB use by ticking the corresponding day for adherence monitoring. The logbook will be reviewed at every visit to assess compliance.

### Procedures and Follow-Up Schedule

Participants will attend 4 visits: baseline (T0), week 1 (T1), month 1 (T2), and month 3 (T3).

#### Clinical Assessments (At All Visits)

The oral examination in accordance with clinical practices will be carried out by a dentist who will report the following clinical parameters:

1. BoP: dichotomous gingival index (GI) reporting the presence or absence of BoP after 30 seconds (0=absence of bleeding after 30 s and 1=presence of bleeding after 30 s). A total of 4 sites are recorded per tooth (mesiobuccal, distobuccal, mesiopalatine, and distopalatine) [14,15].
2. Gingivitis score (GI): measured by visual observation from 0 to 3 (0=no inflammation; 1=slight change, slight inflammation in color, and little change in texture; 2=moderate, moderate inflammation, redness, edema, and hypertrophy, tendency to bleed on probing; 3=severe, marked redness, inflammation, hypertrophy, and tendency to spontaneous bleeding). Gingivitis score=sum of GI scores divided by the number of total sites [16].
3. Dental plaque score (Rustogi Modified Navy Plaque Index): according to the Navy Plaque Index modified by Rustogi et al [17], the presence of plaque deposits is sought on the vestibular and lingual surfaces of the teeth. Each tooth face is divided into 9 areas to which a dichotomous score is assigned (0=absence of plaque and 1=presence of plaque). Thus, for each tooth, 18 measurements are carried out. This index makes it possible to detect minimal differences at the partial level of the marginal or interproximal zones, or at the total level of the oral cavity [17].
4. PPD score: a measure indicating the distance separating the top of the marginal gingiva from the bottom of the periodontal pocket. The measurement is expressed in millimeters. Overall, 4 sites are recorded per tooth (mesiobuccal, distobuccal, mesiopalatine, and distopalatine) [18].
5. CAL score: addition of PPD and recession height, which is the distance separating the enamel-cementum junction from the bottom of the pocket. In total, 4 sites are recorded per tooth (mesiobuccal, distobuccal, mesiopalatine, and distopalatine) [15].
6. Interdental bleeding: bleeding was assessed dichotomously following interdental brushing (0=absence of bleeding after insertion and removal of the brush and 1=presence of bleeding after insertion and removal). All interdental sites were recorded at each visit.

PPD and CAL will be assessed following the criteria defined by the consensus report of the 2017 World Workshop on the Classification of Periodontal and Peri-Implant Diseases and Conditions [13], using the US Williams PDT sensor probe (Zila-Pro-Dentec Inc) at a pressure of 20 g. Based on severity-defined stages of interdental CAL and tooth loss, complexity, extent, and distribution, they included a full oral examination of 6 sites on each permanent tooth. All measures will be performed on all teeth except for third molars. One calibrated dentist trained will perform the periodontal evaluations of all participants. Intraclass correlation coefficients for PPD and CAL will be determined at the level of the site. The intraexaminer coefficients for CAL will be, respectively, from 0.80 and 0.85, and from 0.75 and 0.85 for probing depth.

#### Interdental Microbiota Sampling (T0 and T3)

Interdental plaque will be collected from 4 sites (15-16, 25-26, 35-36, and 45-46). Each IDB used for sampling will be stored in RNase- and DNase-free tubes at –20°C within 4 hours and transferred later to the GenEPI platform (Hospices Civils of Lyon, Lyon, France) for long-term storage at −80°C. Analysis will be performed by 16S ribosomal RNA gene sequencing to determine microbiota composition in a single batch. Briefly, a 16S ribosomal RNA gene fragment comprising V3 and V4 hypervariable regions will be amplified using an optimized and standardized 16S amplicon library preparation. Sequencing will then be performed using a 250-base pair paired-end sequencing protocol on an Illumina MiSeq platform (Illumina). Bioinformatic analysis will then be performed using the QIIME2 software (Caporaso Lab, Northern Arizona University) in its latest version.

#### Questionnaires

Participants will complete 2 questionnaires: (1) First, the baseline oral hygiene questionnaire at T0 (Multimedia Appendix 1) that analyzes habits, frequency, and duration of daily hygiene; and (2) second, the Theoretical Framework of Acceptability (TFA) administered at T1, T2, and T3 (Multimedia Appendix 2). The TFA includes dimensions of perceived effort, intervention coherence, affective response, perceived effectiveness, and self-efficacy, allowing assessment of feasibility and long-term integration. The trial participation schedule is detailed in Table 2.

**Table 2.** Trial participation schedule.

Procedures and visits	Timeline			
	T0 (baseline)	T1 (1 week / T0)	T2 (1 month / T0)	T3 (+3 months / T0)
Prescreening	✓			
Eligibility screening	✓			
Informed consent	✓			
Questionnaires	✓	✓	✓	✓
Oral hygiene attitudes and practices**^a^**	✓			
Acceptability of IDB^b^^c^		✓	✓	✓
Oral examination	✓	✓	✓	✓
BoP^d^	✓	✓	✓	✓
Interdental bleeding	✓	✓	✓	✓
GI^e^	✓	✓	✓	✓
Plaque index	✓	✓	✓	✓
PPD^f^	✓	✓	✓	✓
CAL^g^	✓	✓	✓	✓
Interdental microbiota sampling	✓			✓

a Multimedia Appendix 1 lists oral hygiene attitudes and practices.

b IDB: interdental brush.

c Multimedia Appendix 2 lists the acceptability of interdental brush.

d BoP: bleeding on probing.

e GI: gingival index.

f PPD: probing pocket depth.

g CAL: clinical attachment loss.

### Outcomes

#### Primary Outcome Measures

Reduction in interdental inflammation, expressed as the proportion of sites with BoP at 3 months (T3), comparing calibrated versus noncalibrated IDBs.

#### Secondary Outcome Measures

Secondary outcome measures will be (1) biological outcomes: composition of interdental microbiota (16S ribosomal RNA sequencing, T0 and T3); (2) clinical outcomes: evolution of plaque index, GI, BoP, PPD, and CAL at T1, T2, and T3; and (3) patient-reported outcomes: acceptability and integration of daily interdental cleaning, assessed by the TFA questionnaire at T1, T2, and T3.

### Sample Size

The reduction in gingival bleeding was considered the primary outcome variable, and an estimate of the mean difference in reduction was used to calculate the sample size. Based on a 2-sided α of .05, 80% power, an intraclass correlation of 0.8, and an expected 25% difference in BoP between groups, a total of 50 participants (200 sites) are required. Taking into account a dropout rate of 10%, in order to have 50 participants at the end, 56 participants (28 per group) will be included.

### Recruitment

Participants will be recruited via an existing database of healthy volunteers previously enrolled in oral health studies at the P2S laboratory (Université Lyon 1) and public announcements on social media and digital platforms of the laboratory. All participants will receive free study materials (IDBs for 3 months) and reimbursement of travel costs (2 local transportation tickets per visit).

### Randomization and Blinding

#### Randomization

Participants will be randomly assigned to one of 2 arms (arm 1: calibrated IDBs and arm 2: noncalibrated IDBs). Randomization will be performed using an interactive web response system integrated into the electronic case report form (v8.2.30 build 3, EnnovClinical Group). Allocation will be 1:1 without stratification, as the sample size is limited and the population is considered homogeneous. Participants will remain in the same arm during the study period. A total of 50 participants will be enrolled, with 25 assigned to each group.

#### Blinding

The study will be conducted under single-blind conditions. Participants will not know whether they are assigned to the calibrated or noncalibrated IDB group. To maintain blinding, both types of IDBs will be identical in packaging, color, and appearance. The investigator responsible for the allocation will not be blinded. Intervention implementation will be conducted by staff independent from data collection. To ensure impartiality, during the assessment and analysis phase, statisticians, clinical research associates, and clinicians will not know the group to which the participant belongs. The identification codes will be securely held by the study monitor and will remain sealed until the conclusion of the study to maintain confidentiality and prevent bias.

### Data Collection, Management, and Analysis

#### Data Collection

A case report form, specific to the HIJA study, will be created in Lyon, France. Only data required for the protocol and scientific publication will be recorded in the case report form. Each participant will be assigned a unique identification code to label all documents, forms, and data files. Thus, data recorded by investigators for each participant will be anonymized according to the Data Protection Act. As data are collected, they must be completed by authorized persons (investigators) with their own identifiers, according to the Data Protection Act. During the entry, the data are immediately checked thanks to consistency checks. The person in charge of filing must validate and justify any value change in the electronic case report form. Entries and modifications are subject to an audit trail. The quality, exactitude, and relevance of all input data will be the responsibility of the investigator. Accordingly, each page of the patient’s must be electronically dated and signed by the investigator to confirm agreement and responsibility for the collected data.

#### Data Management

Experimental and personal data will be analyzed using a unique identification code with no directly identifiable personal data. Participants can be reidentified through a subject identification file, which will be securely maintained solely at the clinical study site. The signed informed consent documents will also be kept exclusively at the clinical study site for confidentiality and privacy purposes.

#### Data Analysis

Statistical analyses will be conducted on both the intention-to-treat and per-protocol populations. All randomized participants will be included in the intention-to-treat analysis, while the per-protocol analysis will exclude those with major protocol deviations, such as nonuse of IDBs. Missing data will be addressed using multiple imputation by chained equations with Monte Carlo Markov Chain methods, assuming data are missing at random. Descriptive statistics, including means, SDs, medians, interquartile ranges, and frequencies, will be calculated. Between-group comparisons will be performed using Student 2-tailed *t* test or the Wilcoxon test for continuous variables and the Chi-square test for categorical variables. Changes over time will be assessed using mixed linear models, with log-transformed bleeding values when appropriate. Fixed effects will include independent variables such as age, socioeconomic status, and experimental group, while subjects will be included as a random effect to account for repeated clinical bleeding measurements. Estimated coefficients for each independent variable will be tested against the null hypothesis of zero, with significance set at *P*<.05. Analyses will be performed using SPSS (IBM Corp), SUDAAN (RTI International), and R software (R Development Core Team). Exploratory analyses will also be performed to assess potential correlations between CAL and participants’ adaptation and compliance with IDB use, as evaluated through the TFA questionnaire and the compliance logbook.

### Ethical Considerations

The HIJA protocol was approved by ethical and regulatory authorities and will be performed in conformity with the Declaration of Helsinki. The Committee for the Protection of Persons Sud-Méditerranée I (Marseille, France) approved the protocol on May 14, 2025 (2024-A02813-44 - 69HCL24_1272). This study will be conducted in accordance with the methodology of reference MR-001 of the National Commission for Information Technology and Liberties. This trial was registered with ClinicalTrials.gov (NCT06848790). Before taking part in the study, all participants must provide written informed consent. The following elements will be included in the consent form: (1) the name and affiliation of the principal investigator, (2) a clear description of the purpose of the study, (3) the procedure and duration of the study, (4) the right to stop at any time, (5) the approval of the ethics committee, and (6) a confidentiality guarantee. No compensation will be provided to participants

## Results

The HIJA trial is designed to address a critical gap in current oral health research, the comparative effectiveness of calibrated versus noncalibrated IDBs in daily practice among young adults. While IDBs are established as the most effective interdental cleaning method, clinical evidence is still lacking on whether calibration, a simple yet potentially impactful procedure, provides measurable additional benefits in reducing interdental inflammation and maintaining a balanced oral microbiota. Most available studies have assessed IDBs without clearly distinguishing calibrated from noncalibrated devices, which constitutes an important methodological weakness. This lack of differentiation limits the strength of current recommendations and contributes to heterogeneity in clinical practice. Calibration with a colorimetric probe theoretically optimizes the fit between the brush and the interdental space, thus enhancing mechanical efficacy while minimizing discomfort or trauma [10]. However, in real-life settings, calibration is often overlooked, mainly for reasons of simplicity, time, or cost, raising the question of whether its omission compromises clinical outcomes.

## Discussion

### Principal Findings

The HIJA trial is expected to generate high-quality evidence on whether calibration of IDBs offers a measurable clinical advantage. Because participants’ comfort and adherence may influence interdental cleaning efficacy, compliance will be systematically assessed using the TFA questionnaire and the compliance logbook. These measures will allow the analysis of whether differences in adherence between groups could act as confounding factors in the interpretation of clinical outcomes. If calibration proves beneficial, it could support the systematic implementation of calibrated interdental cleaning in preventive dentistry, particularly in young adults at the onset of lifelong oral hygiene routines. Furthermore, integration of microbiological profiling will enhance the understanding of how interdental cleaning strategies shape the oral microbiome, which may contribute to broader preventive approaches against systemic diseases associated with periodontal inflammation. Ultimately, this research could provide an evidence base for updating oral hygiene guidelines and promoting personalized preventive strategies in dentistry. Another major strength of the HIJA trial is that it will be the first study to perform sequencing of the interdental microbiota in the context of a randomized clinical trial. This innovative approach will allow the exploration of whether calibrated versus noncalibrated IDBs differentially influence microbial composition and oral ecological balance, thereby providing novel mechanistic insights beyond conventional clinical indices. By combining clinical, microbiological, and acceptability outcomes, the HIJA trial will clarify whether calibration provides a meaningful advantage in reducing interdental inflammation and improving oral health. The results may ultimately inform preventive strategies and contribute to the refinement of oral hygiene recommendations in early adulthood.

### Conclusions

By combining clinical, microbiological, and acceptability outcomes, the HIJA trial will clarify whether calibration provides a meaningful advantage in reducing interdental inflammation and improving oral health. The results may ultimately inform preventive strategies and contribute to the refinement of oral hygiene recommendations in early adulthood.

## Supplementary material

10.2196/85154 Multimedia Appendix 1 Oral hygiene questionnaire.

10.2196/85154 Multimedia Appendix 2 Theoretical framework of acceptability.

## Abbreviations

BoP: bleeding on probing
CAL: clinical attachment loss
GI: gingival index
HIJA: Hygiene of Interdental Junctions in Adults
IDB: interdental brush
PPD: probing pocket depth
SPIRIT: Standard Protocol Items Recommendations for Interventional Trials
TFA: Theoretical Framework of Acceptability
