# Supplementary material for: Effectiveness of Different Methods of Interdental Hygiene in Daily Practice Among Young Adults: Protocol for a Randomized, Single-Blind Controlled Trial
Source: JMIR Res Protoc. 2025 Dec 4;14:e85154. doi: 10.2196/85154 (PMC12677869; doi:10.2196/85154)
Supplement: Multimedia Appendix 1 [file resprot-v14-e85154-s001.docx]

**Oral hygiene questionnaire**

**1. How many times do you brush your teeth per day (number of times/day)?**

1 O 2 O 3 O More than 3 O

**2. How long do you currently brush your teeth (brushing time in minutes)?**

Less than 1 minute O 1 to 2 minutes O 3 to 5 minutes O More than 5 minutes O

**3. What kind of toothbrush do you use?**

Manual O Electric O Both O

**4. What type of toothbrush head do you use?**

Hard O Medium O Soft O Ultra soft O Don't know O

**5. How often do you use...?**

|  | Often | Occasionally | Rarely | Never | Don’t know |
| --- | --- | --- | --- | --- | --- |
| Toothpaste | O | O | O | O | O |
| Fluoride toothpaste | O | O | O | O | O |
| Mouthwash | O | O | O | O | O |
| Fluoride mouthwash | O | O | O | O | O |
| Antiseptic mouthwash | O | O | O | O | O |
| Dental floss | O | O | O | O | O |
