# Supplementary material for: Effectiveness of Different Methods of Interdental Hygiene in Daily Practice Among Young Adults: Protocol for a Randomized, Single-Blind Controlled Trial
Source: JMIR Res Protoc. 2025 Dec 4;14:e85154. doi: 10.2196/85154 (PMC12677869; doi:10.2196/85154)
Supplement: Multimedia Appendix 2 [file resprot-v14-e85154-s002.docx]

**Table S1.**

| Did you like or dislike interdental brushes?   \| Dislike \| Don’t like \| No opinion \| Like \| Like a lot \| \| --- \| --- \| --- \| --- \| --- \| \| 1 \| **2** \| **3** \| **4** \| **5** \|   ***OR***  How comfortable did you feel using interdental brushes?   \| Very uncomfortable \| Uncomfortable \| No opinion \| Comfortable \| Very comfortable \| \| --- \| --- \| --- \| --- \| --- \| \| 1 \| **2** \| **3** \| **4** \| **5** \| |
| --- | --- | --- | --- | --- | --- | --- | --- | --- | --- | --- | --- | --- | --- | --- | --- | --- | --- | --- | --- | --- |
| How much effort was required to use the interdental brushes?   \| No effort at all \| A little effort \| No opinion \| Some effort \| A lot of effort \| \| --- \| --- \| --- \| --- \| --- \| \| 1 \| **2** \| **3** \| **4** \| **5** \| |
| Are there any moral or ethical consequences to using interdental brushes?   \| Strongly disagree \| Disagree \| No opinion \| Agree \| Strongly agree \| \| --- \| --- \| --- \| --- \| --- \| \| 1 \| **2** \| **3** \| **4** \| **5** \| |
| Using interdental brushes has improved my oral hygiene:   \| Strongly disagree \| Disagree \| No opinion \| Agree \| Strongly agree \| \| --- \| --- \| --- \| --- \| --- \| \| 1 \| **2** \| **3** \| **4** \| **5** \| |
| It is clear to me how using interdental brushes will help me improve my dental hygiene.   \| Strongly disagree \| Disagree \| No opinion \| Agree \| Strongly agree \| \| --- \| --- \| --- \| --- \| --- \| \| 1 \| **2** \| **3** \| **4** \| **5** \| |
| How confident did you feel about committing to using interdental brushes for?   \| Not at all confident \| Confident \| No opinion \| Confident \| Very confident \| \| --- \| --- \| --- \| --- \| --- \| \| 1 \| **2** \| **3** \| **4** \| **5** \| |
| Using interdental brushes interferes with my other priorities.   \| Strongly disagree \| Disagree \| No opinion \| Agree \| Strongly agree \| \| --- \| --- \| --- \| --- \| --- \| \| 1 \| **2** \| **3** \| **4** \| **5** \| |
| How acceptable was the use of interdental brushes for you?   \| Totally unacceptable \| Unacceptable \| No opinion \| Acceptable \| Fully acceptable \| \| --- \| --- \| --- \| --- \| --- \| \| 1 \| **2** \| **3** \| **4** \| **5** \| |
